# Comparative Evaluation of Machine Learning Models for Residential PM_1_ Prediction in Zagreb (Croatia): Identifying Key Predictors and Indoor/Outdoor Dynamics

**DOI:** 10.3390/toxics14040299

**Published:** 2026-03-29

**Authors:** Marija Jelena Lovrić Štefiček, Silvije Davila, Gordana Pehnec, Ivan Bešlić, Željka Ujević Andrijić, Ivana Banić, Mirjana Turkalj, Mario Lovrić, Luka Kazensky, Goran Gajski

**Affiliations:** 1 Institute for Medical Research and Occupational Health, 10000 Zagreb, Croatia; mlovric@imi.hr (M.J.L.Š.);; 2 Faculty of Chemical Engineering, University of Zagreb, 10000 Zagreb, Croatia; 3 Srebrnjak Children’s Hospital, 10000 Zagreb, Croatia; 4 The School of Medicine, Catholic University of Croatia, 10000 Zagreb, Croatia; 5 Faculty of Medicine, University of J. J. Strossmayer, 31000 Osijek, Croatia; 6 Center for Applied Bioanthropology, Institute for Anthropological Research, 10000 Zagreb, Croatia; 7 Lisbon Council, 1000 Brussels, Belgium

**Keywords:** indoor air quality, outdoor pollution, particulate matter, regression, machine learning, SHAP, public health

## Abstract

Indoor exposure to particulate matter (PM) is increasingly recognized as a major contributor to respiratory and cardiovascular risk, yet the relative contributions of outdoor pollution, building characteristics, and occupant behavior remain poorly resolved. PM_1_ (aerodynamic diameter < 1 μm) warrants focus due to its higher alveolar deposition. “Evidence driven indoor air quality improvement” (EDIAQI) project aims to enhance indoor air quality guidelines and increase awareness by providing accessible data on exposure, pollution sources, and related risk factors. As part of the Zagreb pilot within the project, 103 paired indoor/outdoor PM_1_ samples were analyzed. Seasonal analysis revealed substantial wintertime outdoor PM_1_ spikes, while indoor medians remained stable. Chemometric analysis identified factors such as dwelling size, outdoor pollution, resuspension, building age/heating type, and urban context. Among the tested models, the validated gradient-boosted regressor (GBR) achieved the strongest performance, explaining ~65% variance in indoor PM_1_ (test R^2^ ≈ 0.65). Explainable machine learning analysis (SHAP) identified outdoor PM_1_ levels, infiltration, and resuspension as the most influential predictors. Findings underscore wintertime outdoor emissions (e.g., residential heating and traffic) and dwelling-related and behavioral factors as key drivers, with the machine learning–environmental data integration enabling targeted residential IAQ management: optimized ventilation protocols, resuspension mitigation via behavior, and infiltration reduction through retrofits.

## 1. Introduction

Indoor air quality (IAQ) has emerged as a critical factor in environmental health because people in high-income countries now spend approximately 70–90% of their time in enclosed spaces such as homes, schools, and workplaces [1,2]. Residential buildings are particularly important microenvironments, as they accommodate vulnerable populations, and their IAQ reflects an interplay of geography, building type, ventilation design, and occupants’ daily activities [1]. A large body of epidemiological and toxicological evidence identifies particulate matter (PM) as one of the predominant air pollutants affecting human health globally, with fine PM among the main contributors to premature mortality and reduced life expectancy [2,3,4,5]. Since a substantial fraction of total exposure occurs indoors, especially in residential settings, indoor PM pollution has become a key focus for public health risk assessment and mitigation [6,7,8,9]. Globally, the World Health Organization (WHO) identifies air pollution, driven primarily by PM, as the single largest environmental health risk and a leading cause of mortality. Ambient and household air pollution together are estimated to cause roughly 7 million premature deaths annually, with household (indoor) pollution accounting for about 3.8 million [9,10]. WHO’s current air quality guidelines [11], as well as regulatory air quality standards, continue to focus on mass-based PM_10_ and PM_2.5_ metrics. However, due to the growing evidence that smaller fractions such as PM_1_ and ultrafine particles (UFPs) pose particularly severe health risks [12,13], their measurements are strongly encouraged.

IAQ in residential buildings is shaped by the interaction of outdoor air quality, occupant behavior, and building characteristics [14]. Outdoor pollutant concentrations and building airtightness govern the extent to which ambient PM infiltrates indoors, with ventilation rate, pollutant lifetimes, and indoor-to-outdoor mixing ratios controlling how strongly indoor levels track outdoor conditions [14,15,16,17]. In many settings, outdoor pollutant concentrations are the dominant driver of indoor concentrations [18,19]. However, there are also situations in which indoor pollutant levels exceed outdoor levels, challenging traditional assumptions and underscoring the importance of indoor sources and microenvironment-specific processes [20]. Occupant presence and activities constitute a major source of indoor PM, often amplifying outdoor contributions. Across indoor environments, higher occupancy systematically increases PM through direct human emissions, resuspension of settled dust, intensified movement, and activity-related sources such as cooking, smoking, and cleaning [21,22,23,24,25]. Construction materials, ongoing renovations, and building geometries (e.g., large open rooms, floor level close to traffic, and façade orientation) further modulate PM levels by altering emission sources, resuspension dynamics, and exposure to outdoor PM [26].

Particle size largely determines respiratory deposition, systemic distribution, and toxicity. Fine particles (PM_2.5_) and smaller fractions (PM_1_ and UFPs) can efficiently bypass the upper airways, deposit deep in the bronchioles and alveoli, and, in the smallest size ranges, cross the air–blood barrier into the bloodstream [2,27,28]. Toxicological studies show that smaller particles, per unit mass, exhibit greater oxidative and pro-inflammatory potency because their high surface-area-to-volume ratio allows them to carry more redox-active components, such as transition metals and polycyclic aromatic hydrocarbons (PAHs), thereby catalyzing the formation of reactive oxygen species (ROS), oxidative DNA damage, and mitochondrial dysfunction [28]. Consistent with these mechanisms, smaller particles are linked not only to respiratory outcomes (e.g., asthma and chronic obstructive pulmonary disease) but also to cardiovascular events, diabetes, neurodevelopmental and neurodegenerative disorders, and systemic inflammation, while long-term exposure is associated with increased mortality from ischemic heart disease, stroke, and cancer [3,12,27,29,30,31,32,33,34,35].

Recent research indicates that PM_1_ can generate a health burden comparable to that of PM_2.5_. In one study, PM_1_ accounted for 4.47% of emergency visits, nearly matching the 5.05% attributable to PM_2.5_, despite PM_1_ being a subset of PM_2.5_ mass [36].

In this study, the PM_1_ fraction, in particular, has been selected because it allows investigation of particle exposures and associated health risks that are not fully captured by the more commonly studied PM_2.5_ and PM_10_ metrics. While routine outdoor monitoring of PM_2.5_ and PM_10_ has enabled the establishment of associations with health outcomes and informed guideline values, including those proposed by the WHO [11], finer particles such as PM_1_ and ultrafine particles remain considerably less studied. PM_1_ particles are small enough to penetrate deep into the alveolar region, cross cellular membranes, and enter the bloodstream, which may increase the risk of adverse health effects. Indoor air can be dominated by combustion-related PM_1_ particles from sources such as cooking, frying, grilling, smoking, and candles, making PM_1_ a sensitive indicator of recent combustion activity [37]. Outdoor sources of PM_1_, such as traffic, can contribute more to indoor pollution than larger particles due to their higher infiltration capability. Previous research in the studied area has shown that PM_1_ in ambient air often represents a large proportion of the PM_2.5_ fraction, which also refers to the many carcinogenic PM-bound compounds. Their PM_1_/PM_2.5_ ratio was about 80% during the winter months [38], sometimes exceeding 90% for individual PAHs [39]. Preliminary studies of PAHs and polybrominated diphenyl ethers (PBDEs), carried out in a smaller number of households, have also shown their presence in the PM_1_ particle fraction [40,41]. These characteristics justify the focus on PM_1_ in the present study and address the existing knowledge gaps regarding fine particle exposures and their health implications.

Accurate characterization of indoor PM concentrations and temporal dynamics is essential for both exposure assessment and intervention design, yet it remains methodologically challenging. Regulatory monitoring is traditionally grounded in gravimetric filter-based reference methods, which provide accurate 24 h mass concentrations, enable subsequent chemical analysis, and serve as the basis for standards and long-term trend analyses [42,43]. However, these methods are noisy, expensive, and ill-suited to occupied indoor environments. Real-time optical monitors and low-cost sensors can resolve fine-scale temporal patterns but exhibit biases that depend on particle size, composition, relative humidity, and other factors, creating the need for setting-specific calibration against gravimetric reference instruments [27,44]. These measurement constraints contribute to the scarcity of large-scale, long-term indoor PM datasets and complicate efforts to link IAQ to health outcomes.

Meteorological and climatic conditions strongly influence both outdoor PM and its indoor manifestations. In continental regions characterized by flat terrain and strong cold–hot contrasts, such as inland Croatia, statistical models show that PM_2.5_ levels are closely linked to thermal and land surface variables that reflect residential heating, stable cold layers, and urban structure, all of which favor pollution build-up under inversion conditions [45]. Winter temperature inversions and associated high-pressure, weak-mixing regimes can lead to more than 10-fold higher PM_1_-bound PAH concentrations in the cold season compared with warmer months, when stronger mixing and higher wind speeds promote dilution and advection of cleaner air masses [46]. At the building scale, seasonal meteorology modulates indoor-to-outdoor gradients. In winter, strong negative correlations between wind speed and PM (especially PM_1_) and negative associations with minimum temperature indicate that cold, anticyclonic conditions favor pollutant accumulation, while large indoor–outdoor temperature differences enhance the “chimney effect” and more tightly couple indoor PM to outdoor concentrations. In summer, smaller or reversed temperature gradients can produce a “reverse chimney effect,” whereby infiltration pathways change and indoor PM remains elevated even when outdoor levels are relatively low [47].

With the aim of finding relationships between indoor and outdoor pollutant concentrations, meteorological parameters, outdoor environment, indoor occupancy, and building characteristics, recent research has increasingly focused on predictive modeling of indoor PM as a complement to, or alternative to, direct monitoring. For example, machine learning (ML) and hybrid approaches have been proposed to estimate indoor PM_2.5_ using outdoor concentrations, building characteristics, meteorological parameters, and limited IAQ measurements, with a goal of providing generalized, easy-to-use tools for population-level exposure assessment [6,7,48,49,50]. Even in the more data-limited settings, typical of single-city or single-station studies, relatively simple regression methods can achieve satisfactory performance with modest computational cost, enabling broader uptake in applied IAQ and building environment research [51]. A wide range of supervised models has been deployed for IAQ and indoor PM applications, including linear regression, decision trees, Random Forest, support-vector regression, k-nearest neighbors, gradient-boosted trees, and neural architectures such as LSTMs [9,12,52].

Comparative studies using diverse regressors on sensor data show that calibration and prediction performance are strongly sensor- and pollutant-dependent rather than algorithm-agnostic, highlighting the need to test multiple model classes for each specific deployment. In outdoor AQ forecasting and post-processing of deterministic models, ensemble and boosting algorithms such as Random Forest (RF), XGBoost, LightGBM, and CatBoost consistently demonstrate strong performance. RF is often the dominant model overall, while boosted trees or hybrid and deep learning approaches can excel in specific cases or tasks [53]. Despite these advances, recent reviews emphasize persistent challenges in indoor applications, including small and location-specific datasets, inconsistent labels, limited representation of rare events, sensor variability, and weak integration of physical and chemical knowledge. These factors continue to constrain model transferability across building types and hinder robust uncertainty quantification [54].

In the IAQ domain specifically, ML techniques have been applied to address a range of tasks, including (i) calibration of low-cost PM sensors in nurseries, schools, and other indoor environments, (ii) prediction of indoor pollutant time series, and (iii) modeling of key IAQ indicators such as indoor PM_2.5_, CO_2_, and airflow from limited sensor inputs [9,55,56,57,58,59]. For instance, gradient-boosted tree models have been used to link occupancy, area per person, outdoor meteorological variables, and Air Quality Index (AQI) with indoor CO_2_ concentration, as well as to jointly predict temperature and humidity. These studies demonstrate that ML models can operate effectively within building-scale monitoring networks and smart building control systems [60,61]. In residential and complex building settings, boosted ensemble models such as CatBoost have shown strong predictive power for identifying indoor PM_2.5_ exceedance events, suggesting that such exceedances arise from intricate interactions among outdoor PM levels, meteorological factors, occupancy patterns, and building characteristics that are effectively captured by tree-based approaches [62,63]. However, most previous studies have focused on relatively isolated modeling objectives or single pollutant-specific analyses, while integrated frameworks that jointly consider physical, environmental, and human-related factors remain scarce. Further research is therefore needed to develop structured, comprehensive modeling strategies, an aspect that is addressed in this study.

This research was carried out within the framework of the project “Evidence driven indoor air quality improvement” (EDIAQI) [64], which aims to enhance IAQ guidelines and increase awareness across Europe and beyond by providing accessible data on exposure, pollution sources, and related risk factors. The Zagreb (Croatia) pilot study, as part of EDIAQI, aims to evaluate the effects of indoor and outdoor air pollution on children’s health. It employed advanced monitoring techniques and biological sampling to investigate the links between IAQ, health outcomes, and environmental factors in residential environments.

This study represents the first comprehensive investigation of indoor PM_1_ pollution in this region, combining direct measurements, questionnaire data, publicly available datasets, and advanced statistical and machine learning (ML) tools. The research is based on parallel gravimetric indoor and outdoor PM_1_ measurements in residential households in Zagreb City and Zagreb County, and includes: (i) reporting indoor PM_1_ mass concentrations from more than 100 households, which are currently the only such data available for this part of Europe; (ii) investigating seasonal and indoor–outdoor differences in PM_1_ concentrations; (iii) exploring the relationships between indoor and outdoor PM_1_, household characteristics, occupant activities, and environmental and land use indicators using advanced statistical methods; and (iv) identifying variables of significance for predictive ML models.

The novelty of this study lies in its structured analytical approach, which integrates process-based chemometric analysis, principal component analysis (PCA), feature engineering, supervised ML, and SHapley Additive exPlanations (SHAP) interpretation toward addressing the main research objective. To the best of our knowledge, research that combines traditional approaches, supervised ML with SHAP analysis to interpret residential PM_1_ concentrations in Central and Southeastern Europe remains scarce. By addressing this gap, our study provides new insights into the determinants of indoor PM_1_ pollution and demonstrates the utility of modern analytical approaches for exposure assessment and risk evaluation.

## 2. Materials and Methods

### 2.1. Data Collection

**Figure 1:**
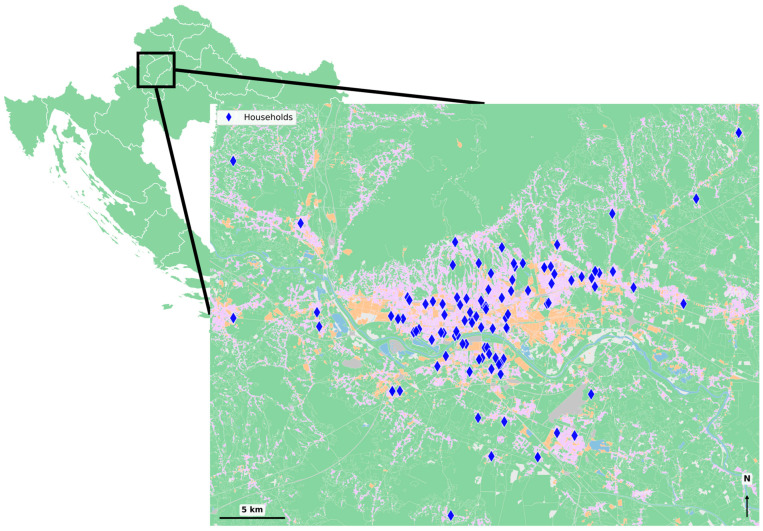
Measurement locations across Zagreb City and Zagreb County, Croatia (blue diamonds—sampling sites; urban area—purple; roads—grey; industrial/commercial area—orange; green/nature—green; water—blue).

Within the scope of the Zagreb pilot within the “Evidence driven indoor air quality improvement” (EDIAQI) project, the indoor and outdoor PM_1_ (particulate matter with an aerodynamic diameter below 1 μm) samples were collected in the participant households on filters through active sampling using pumps over a 7-day (~168 h) period. Conventional low-volume reference samplers typically used for outdoor or ambient air monitoring (operating at approximately 55 m^3^ day^−1^) are unsuitable for indoor use because of their large size and high noise levels. Instead, we employed smaller, quieter devices (Sven-Leckel MiniVS-C, Berlin, Germany, and MiniVol Portable Air Sampler, AirMetrics, Eugene, OR, USA; 5 L min^−1^), which are specifically suited for week-long indoor deployments and, when used over 7 days, provide adequate sampling volumes for reliable filter-based PM_1_ analysis in this residential context. These instruments were fitted with a size-selective impactor inlet targeting the PM_1_ fraction of particulate matter and a filter holder containing quartz filters (Whatman, Tisch Scientific, Cleves, OH, USA). The samplers were placed in residential bedrooms or living rooms of households across the Zagreb City (767,131 residents) and Zagreb County (299,985 residents) [65] with regard to uniform coverage of samples in the area (Figure 1 was generated in Jupyter notebook using Copernicus Urban Atlas and approximate dwelling coordinates [66]).

Outdoor PM_1_ samples were collected using identical samplers installed on balconies or terraces directly attached to the participating dwellings, which comprised both apartments and detached/semi-detached houses. The samplers were placed in semi-sheltered positions a few meters from the main façade, typically within a few meters of doors or windows while avoiding immediate proximity to local point sources (e.g., exhaust vents and barbecues). Their placement was designed to capture the dwelling-specific near-outdoor environment rather than distant urban background conditions. Distances to roads and playgrounds, therefore, varied across sites but followed the general residential context of each home. The collected dataset comprised PM_1_ indoor and outdoor concentration data, as well as variables constructed from participants’ questionnaire responses.

### 2.2. Chemometric Analysis

Chemometric analyses were performed on a dataset containing paired indoor–outdoor PM_1_ measurements obtained from residential dwellings. Pollutant metrics included indoor PM_1_, outdoor PM_1_, and their difference (PM_1,diff_ = PM_1,IN_ − PM_1,OUT_). In this study, household and environmental variables were encoded as numerical predictors within the ML framework. Floor area, floor number, and construction year were treated as continuous variables, while renovation status and heating and ventilation system indicators were encoded as ordinal or categorical variables consistent with the questionnaire design. Occupancy, vacuuming frequency, presence of carpets and curtains, presence of tumble-drier, and number of AC units were likewise translated into count, frequency, or binary indicators, ensuring that behavioral and interior surface characteristics could be directly related to indoor PM_1_ levels. Urban land use descriptors (roads, urban, industrial, green) were constructed from the Copernicus Urban Atlas data [66] using the approximate longitude and latitude of dwellings, and they were represented by a compact, multi-level built-form categorical variable. Seasonal conditions were classified into heating and non-heating periods based on the measurement date.

Given the skewed distributions and non-normality typical of environmental concentration data, the analytical workflow relied on non-parametric and multivariate chemometric methods for pattern recognition and variable selection. Before multivariate analysis, all continuous variables were scaled to account for differing measurement units and to ensure balanced feature contributions in subsequent analyses.

### 2.3. Principal Component Analysis (PCA)

Principal component analysis (PCA) is a dimensionality reduction technique that projects the data onto a plane where each coordinate represents a data feature and then transforms the data into a new space where the variation is maximized. Every dataset can have several components, up to the number of features in that dataset, with the first component representing the maximum variation and the last the least [67,68,69,70,71]. The number of retained components was chosen to preserve a high proportion of total variance (approximately 80%) while limiting dimensionality. PCA was applied to identify latent variable domains and to visualize the underlying structure in the dataset. All variables were scaled before analysis. Principal components (PCs) were retained based on inspection of the scree plot and the interpretability of loading patterns. Variables with high absolute loadings (|loading| ≥ 0.2) on a given component were considered influential and used to characterize that component. Principal components were interpreted in terms of indoor-to-outdoor concentration gradients, seasonal variation, urban exposure patterns, and building-related factors, and the loading structure was used to delineate latent domains that subsequently informed variable selection for modeling.

### 2.4. Cluster Analysis

Unsupervised clustering was performed on PCA scores to identify groups of observations with similar sources [72]. A distance-based clustering algorithm was used, with k = 3 clusters chosen based on the silhouette score, visual separation in PCA score plots, and interpretability. Cluster separation was assessed using Mann–Whitney U tests, comparing PM_1_ levels between indoor and outdoor clusters and between clusters with differential PM_1_ levels. Independent Mann–Whitney U tests examined differences between heating and non-heating seasons for indoor PM_1_, outdoor PM_1_, and their difference (PM_1,diff_ = PM_1,IN_ − PM_1,OUT_).

### 2.5. Statistical Software

All analyses were executed in Python 3.12 using the following packages:

- scipy.stats [73] for non-parametric tests (Mann–Whitney U).
- pandas [74] for data management.
- numpy [75] for numerical operations.
- scikit-learn [76] for PCA and clustering.
- matplotlib [77] and seaborn [78,79] for data visualization.

Chemometric methods were employed because environmental exposure datasets are inherently complex: they are typically non-normal, heteroscedastic, and multivariate, with strong inter-variable correlations. The combined application of PCA, clustering, and non-parametric testing enabled the robust identification of latent pollution regimes, seasonal patterns, and indoor-to-outdoor gradients in PM_1_ behavior. Within this framework, PCA primarily served as a structure detection tool to guide feature selection for subsequent ML rather than as a dimensionality reduction step. Variables with the highest loadings on each principal component were interpreted as indicators of distinct latent domains, including dwelling characteristics, ventilation, pollution dynamics, interior surfaces, occupancy, heating systems, and overall building context. To minimize redundancy and multicollinearity while maintaining interpretability, a subset of representative raw variables was selected from each component based on both loading magnitude and conceptual relevance.

### 2.6. Machine Learning (ML)

Machine learning approaches were selected because indoor PM concentrations are influenced by complex and potentially non-linear interactions among outdoor pollution, building characteristics, and occupant behavior. The objective of the ML framework was to predict indoor PM_1_ concentrations from selected predictor variables using supervised regression algorithms. We modeled indoor concentrations in dwellings using a dataset containing outdoor concentrations, building characteristics, land use, and occupant behavior variables selected by chemometric analysis. The data were randomly split into 80% training and 20% test sets (seed = 42).

### 2.7. Feature Engineering

To better reflect the underlying physical processes, we derived a small set of composite predictors from the original variables. Indoor-to-outdoor relationship is highlighted by the truncated I/O ratio $\frac{I}{O}=min\left(1,{PM}_{1,IN}/{PM}_{1,OUT}\right)$, along with its interaction with heating season and the interaction between $PM_{1,\;OUT}$ and heating season. Infiltration effects were summarized by an infiltration index $\left(infiltration\_index=window\_count/floor\_area\right)$, its interaction with PM_1,OUT_, and a floor_number × infiltration to capture vertical variation. Traffic and land use influences were represented by an interaction between outdoor PM_1_ and proximity to major roads. Occupancy and resuspension were described using occupancy density (household members per m^2^), total soft surfaces (carpets + curtains), and a cleaning/resuspension index combining vacuuming frequency with soft surface count. Finally, building age, its interaction with heating season, and a “heating type” category derived from multiple heating system indicators were used to capture building age and heating-related effects.

### 2.8. Pre-Processing and Models

All models shared a common pre-processing pipeline to ensure comparability and cross-validation safety. Predictors were scaled to support stable optimization in gradient-based models [12,80,81]. Based on preliminary performance comparisons, RobustScaler (median and interquartile-range scaling) was chosen. We implemented and compared several commonly used supervised learning algorithms for tabular environmental data, including Decision Tree Regressor (DT), Random Forest Regressor (RF), Gradient Boosting Regressor (GBR), AdaBoost Regressor, Extreme Gradient Boosting (XGB), and CatBoost Regressor. Tree-based ensemble models were chosen due to their ability to balance predictive accuracy with interpretability. Preliminary exploratory runs indicated that tree-based and boosting ensemble models were well-suited to capturing non-linearities and interactions between building, behavioral, and environmental predictors, which motivated their inclusion and subsequent focus in this study. Because PM_1_ was right-skewed, we applied a log-transform to the target using a wrapped regressor with $y^{\prime }=log(1+y)$, an inverse $y=exp(y^{\prime })-1$. The wrapper was included in cross-validation so that tuning and evaluation were performed on the transformed scale, while performance metrics were reported on the original scale.

### 2.9. Hyperparameter Tuning and Model Evaluation

For each algorithm, we performed a hyperparameter search with multi-metric evaluation. Search spaces were regularized (e.g., max_depth 3–6, 100–400 trees, learning rates 0.01–0.1, moderate leaf sizes and strong L2 penalties) to limit overfitting. Cross-validation used repeated k-fold (5 folds × 3 repeats, shuffled, seed = 42). Multiple scoring metrics (negative MSE, negative MAE, R^2^) were computed and models were refit on the configuration, maximizing cross-validated R^2^. The best estimator per algorithm was then retrained on the full training set. Final model performance and generalization was assessed on the held-out test set using the following metrics:

- Mean Absolute Error (MAE).
- Mean Squared Error (MSE).
- Root Mean Squared Error (RMSE).
- Coefficient of Determination (R^2^).

### 2.10. Model Interpretability

Interpretability has become a key requirement as ML outputs are increasingly used to support health-protective decisions and building operation strategies. Tree-based ensembles (RF, XGB, and GBR) are popular partly because they offer built-in measures of feature importance while retaining strong predictive performance [12,82]. Post hoc, model-agnostic tools such as SHapley Additive exPlanations (SHAP) are used to explain complex models without altering their predictive function. To obtain more reliable and consistent attributions, the SHAP values were computed [82,83], providing model-agnostic local and global explanations of feature contributions. SHAP approximates the Shapley values from the cooperative game theory and assigns signed contributions to each feature for each prediction. Aggregating these contributions yields global importance rankings and characteristic directional effects. Global SHAP importance plots and summary (bee swarm) plots were used to interpret dominant predictors and their effect directions on indoor PM_1_, in line with recent applications of explainable ML in air quality research. All implementations were carried out in Python, using scikit-learn for model wrappers, cross-validation, and pre-processing [76], and specialized libraries for XGB [84], CatBoost [85], and SHAP [86], consistent with ML workflows for environmental data analysis.

## 3. Results

### 3.1. Descriptive Statistics of PM_1_, Saeasonal, and Indoor–Outdoor Differences

**Figure 2:**
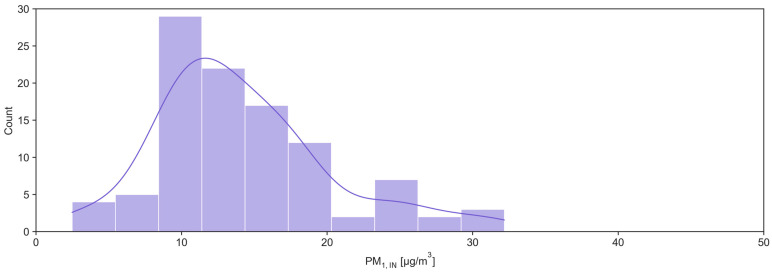
Indoor PM_1_ distribution plot.

**Figure 3:**
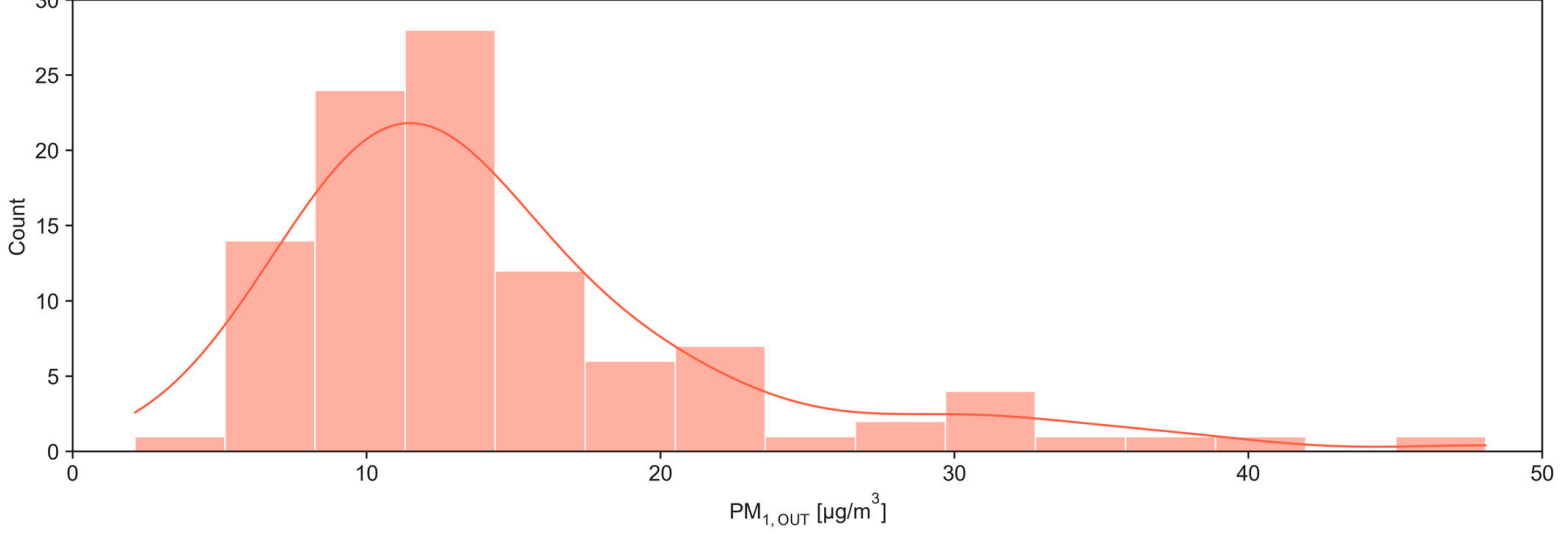
Outdoor PM_1_ distribution plot.

After preliminary analysis and following removal of extreme outliers using the interquartile range (IQR) method, where IQR is defined as the difference between the 75th and 25th percentiles, and observations lying beyond 2 × IQR from the lower or upper quartile are excluded, a total of 103 out of 109 paired indoor and outdoor PM_1_ measurements were retained. Across all measurements, indoor PM_1_ exhibited a slightly right-skewed distribution, with values ranging from 2.48 to 32.17 µg m^−3^ and clustering between roughly 10 and 20 µg m^−3^, whereas outdoor PM_1_ spanned a wider range (2.11–48.07 µg m^−3^), with a more pronounced long tail toward high concentrations (Figure 2 and Figure 3).

Indoor PM_1_ had a slightly higher median overall than outdoor PM_1_ (13.04 vs. 12.56 µg m^−3^), with the median indoor–outdoor median difference (PM_1,diff_) being slightly positive at 0.17 µg m^−3^, although the data show substantial dispersion (|PM_1,diff_| median 3.21 µg m^−3^). The Shapiro–Wilk test for indoor PM_1_ (W = 0.939, *p* = 0.00013) rejected normality, consistent with the visually skewed histogram and supporting the use of non-parametric statistics. The Spearman correlation analysis revealed a moderate positive association between indoor and outdoor PM_1_ concentrations (ρ = 0.48, *p* < 0.001), indicating that outdoor particulate levels contribute measurably but only partially to indoor variability (ρ^2^ ≈ 0.23), with additional indoor and building-related factors likely playing a substantial role. The Spearman correlation did not show an association between heating season and indoor PM_1_, but it indicated a moderate correlation between heating season and outdoor PM_1_ (ρ = 0.45).

Further seasonal analysis (Table 1) revealed that indoor medians were similar between heating and non-heating seasons (13.47 vs. 12.86 µg m^−3^; *p* = 0.41), whereas outdoor PM_1_ was significantly elevated during the heating season (median 15.94 vs. 11.18 µg m^−3^; *p* = < 0.001). Consequently, the indoor-to-outdoor difference inverted. In the non-heating period, PM_1,diff_ was positive (median 1.64 µg m^−3^), while in the heating period, it was negative (median −3.55 µg m^−3^), with both the signed (*p* < 0.001) and absolute differences (*p* < 0.001) significantly larger in the heating versus non-heating (*p* < 0.001) period. The I/O ratio shifted from >1 in the non-heating season (median 1.15) to <1 in the heating season (median 0.80), and the truncated I/O ratio showed the same pattern, indicating stronger outdoor dominance in winter.

### 3.2. Relationship Between PM_1_, Household Characteristics, and Occupant Activities Explored Through Principal Component and Clustering Analysis

**Figure 4:**
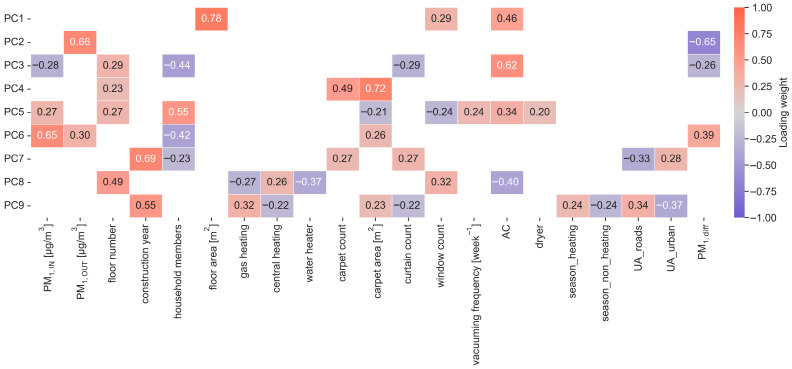
Principal component analysis heatmap of |loading| ≥ 0.2, explaining 80% of variance.

Principal component analysis (PCA) identified nine components that together explained approximately 80% of the total variance in the dataset (Figure 4). PC1 showed strong positive loadings for floor area [m^2^] (0.78), window count (0.29), and air conditioning (AC; 0.46), capturing a dwelling size and ventilation dimension. PC2 was dominated by PM_1,OUT_ (0.66), with a strong negative loading for the indoor-to-outdoor difference PM_1,diff_ (−0.65), reflecting an outdoor pollution gradient that shapes indoor-to-outdoor contrasts. PC3 combined positive loadings for AC (0.62) and floor number (0.29) with negative loadings for PM_1,IN_ (−0.28), household members (−0.44), curtain count (−0.29), and PM_1,diff_ (−0.26), representing a mixed structural and occupancy factor inversely related to indoor concentrations. PC4 and PC5 were primarily associated with interior features: PC4 with carpet area [m^2^] (0.72), carpet count (0.49), and floor number (0.23), and PC5 with PM_1,IN_ (0.27), floor number (0.27), household members (0.55), window count (0.24), AC (0.34), and dryer (0.20), together with negative loadings for carpet area [m^2^] (−0.21) and curtain count (−0.24), indicating structural, furnishing, and appliance use attributes relevant for particle retention and resuspension. PC6 captured a pollution-oriented pattern with positive loadings for PM_1,IN_ (0.65), PM_1,OUT_ (0.30), carpet area [m^2^] (0.26), and PM_1,diff_ (0.39), and a negative loading for household members (−0.42), whereas PC7, PC8, and PC9 were linked mainly to construction year, heating type (central heating or gas heating), season (heating vs. non_heating), and urbanization (UA*).

**Figure 5:**
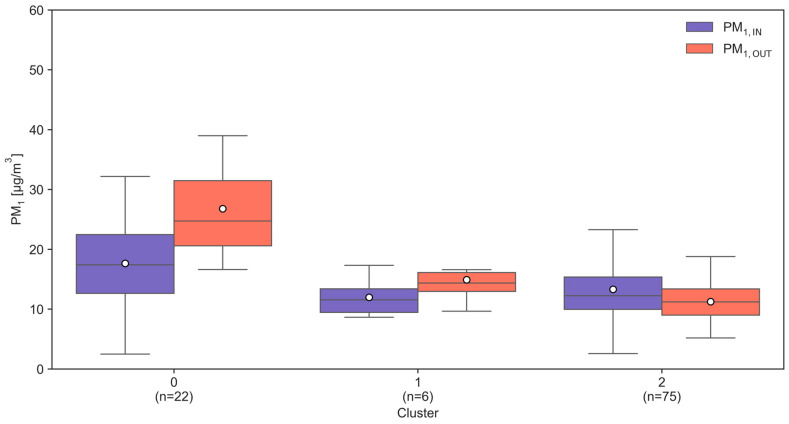
Boxplots of indoor (PM_1,IN_) and outdoor (PM_1,OUT_) PM_1_ concentrations across three clusters with distinct indoor-to-outdoor PM_1_ profiles (median, lower quartile, interquartile range, upper quartile, and minimum and maximum values).

PCA-informed feature selection then focused on variables with strong loadings in each component, yielding a reduced set of raw predictors representing the key latent domains of dwelling structure and ventilation, pollution dynamics, interior surface characteristics, occupancy-related activity, heating systems, and building context. These predictors were used as inputs for subsequent clustering of homes in the principal component space, which separated the sample into three clusters (Figure 5) (*n* = 22, 6, and 75) with distinct indoor-to-outdoor PM_1_ profiles.

Cluster 0 comprised dwellings with the highest overall PM_1_ levels, characterized by elevated indoor and especially outdoor concentrations, with outdoor medians exceeding indoor medians. Cluster 1 showed intermediate PM_1_ levels with relatively similar indoor and outdoor medians, while Cluster 2 was defined by the lowest PM_1_ concentrations in both environments. Mann–Whitney U tests indicated that indoor PM_1_ did not differ significantly between Clusters 0 and 1 (*p* = 0.0517) or between Clusters 1 and 2 (*p* = 0.5198) but was significantly higher in Cluster 0 than in Cluster 2 (*p* = 0.0061). In contrast, outdoor PM_1_ differed significantly across most pairwise comparisons: Cluster 0 versus 1 (*p* = 0.0005), Cluster 0 versus 2 (*p* < 0.0001), and Cluster 1 versus 2 (*p* = 0.0298), indicating that between-cluster separation was driven predominantly by outdoor exposure levels. The differences in the indoor-to-outdoor contrast further emphasized this pattern. The PM_1,diff_ was significantly different between Cluster 0 and 1 (*p* = 0.0143), Cluster 0 and 2 (*p* < 0.0001), and Cluster 1 and 2 (*p* = 0.0300), with Cluster 0 tending toward a larger positive PM_1,diff_.

**Figure 6:**
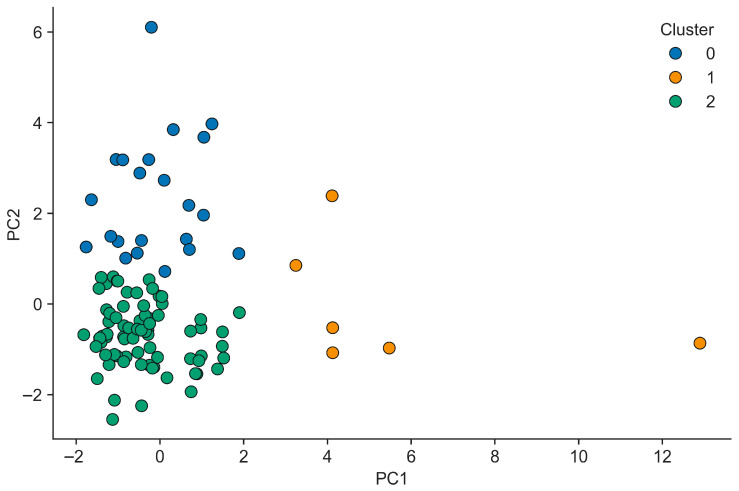
Distinct indoor-to-outdoor PM_1_ profiles visualized in the principal component (PC) space.

When looking into the distinct Cluster 2 containing the majority of households, it can be noted that the indoor PM_1_ concentration is higher than the outdoor concentration (median 12.24 vs. 11.22 µg m^−3^) as opposed to the other two clusters highlighting the contribution of indoor factors alongside outdoor pollution. The visualization of clusters (Figure 6) in the PC1–PC2 space showed Cluster 0 occupying regions associated with larger, better ventilated dwellings but higher overall pollution, Cluster 1 positioned at extreme positive PC1 scores with few observations, and Cluster 2 concentrated near the origin with lower pollution and more moderate structural characteristics.

### 3.3. Optimization and Predictive Capability of Machine Learning Models for PM_1_

Gradient Boosting (GBR), CatBoost, XGBoost, Random Forest (RF), AdaBoost, and Decision Tree (DT) Regressors were tuned using randomized hyperparameter search, yielding compact models tailored to the relatively small sample size.

Across all tested models, gradient boosting (GBR) achieved the best predictive performance, with the lowest test RMSE (4.39) and the highest test R^2^ (0.65), followed closely by CatBoost and XGB (Table 2). Simpler tree-based ensembles (AdaBoost, RF, and DT) showed substantially higher test errors and much lower R^2^ values, indicating a limited ability to capture the non-linear relationships that drive indoor PM_1,_ compared with boosted gradient models.

The tuned GBR model used a moderate learning rate (0.05), 400 estimators, shallow trees (max_depth = 4, min_samples_leaf = 2, min_samples_split = 10), and sub-sampling (subsample = 0.60, max_features = None), consistent with a bias-variance trade-off favoring generalization. The tuned CatBoost model used 300 iterations, depth = 4, learning rate = 0.05, and moderate regularization and stochasticity (bagging_temperature = 0.5, random_strength = 0.5, subsample = 0.8, l2_leaf_reg = 5).

The optimal XGBoost configuration similarly combined a low learning rate (0.01) with 400 trees, max_depth = 5, min_child_weight = 2, and subsample = 0.60, with weak regularization (reg_lambda = 0.10, reg_alpha = 0) and full column sampling (colsample_bytree = 1.0).

While simpler ensembles failed in generalizing on the data, they were also retained to provide interpretable baselines against which to compare the more complex gradient-boosting models. AdaBoost used learning_rate = 0.1 and n_estimators = 200, while Random Forest employed 400 trees of limited depth (max_depth = 5, min_samples_leaf = 2, min_samples_split = 10) with bootstrap sampling and √-rule feature selection and pruned decision tree used max_depth = 4, min_samples_leaf = 4, min_samples_split = 20, ccp_alpha ≈ 0 and max_features = None.

**Figure 7:**
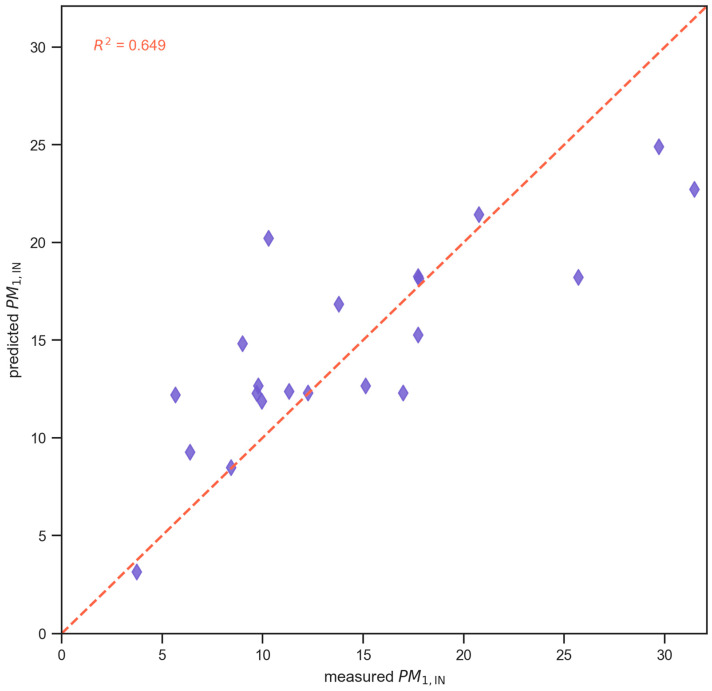
Measured vs. Gradient Boosting Regressor-predicted indoor PM_1_ concentrations.

Figure 7 presents a scatter plot comparing measured and GBR predicted indoor PM_1_ concentrations, with each point representing one observation, a red dashed line indicating the 1:1 perfect agreement line signifying that the model accounts for approximately 65% of the variance in measured PM_1_ with moderate dispersion around the ideal line.

## 4. Discussion

Indoor-to-outdoor PM_1_ relationships in this residential dataset were shaped jointly by season, outdoor pollution, and dwelling characteristics. Overall, indoor and outdoor medians were similar, and the overall PM_1,diff_ was close to zero, indicating that indoor PM_1_ largely tracked outdoor conditions rather than being dominated by persistent indoor sources. Nonetheless, the skewed distributions and large |PM_1,diff_| values indicate substantial variability across homes and seasons, consistent with episodic indoor activities and differing ventilation.

Seasonal analysis revealed stable indoor medians but markedly higher outdoor PM_1_ in the heating season, resulting in an inversion of the indoor-to-outdoor gradient and a shift in I/O ratios from >1 in the non-heating period to <1 in winter. This pattern aligns with reports that winter combustion and reduced dispersion increase outdoor fine particles, so that indoor levels increasingly reflect outdoor infiltration rather than indoor generation. PCA highlighted distinct latent domains: dwelling size/ventilation (floor area, windows, air conditioning), outdoor pollution, interior surface characteristics (carpets, curtains), occupancy and appliance use, building age, heating systems, and urban context [87,88].

Collectively, the chemometric analyses demonstrate that combinations of dwelling structure, ventilation, occupancy, and heating-related factors give rise to distinct exposure profiles, with cluster membership strongly linked to outdoor PM_1_ levels and to the magnitude and direction of the indoor-to-outdoor gradient. In the absence of the established regulatory limit values for PM_1_, the three identified clusters are best understood as empirically derived exposure profiles rather than formal compliance categories. The distributions of indoor and outdoor PM_1_ within each cluster (including the direction and magnitude of the indoor-to-outdoor gradient) inform the development of future guideline frameworks that distinguish between outdoor-dominated and indoor-dominated exposure scenarios, helping to design targeted mitigation strategies such as reducing outdoor particle infiltration or addressing indoor sources and behaviors. The results also show how data-driven profiling, combined with evolving health-based recommendations, can help define provisional, context-specific trigger ranges for residential PM_1_ in future applications. Overall, the evidence suggests that wintertime outdoor pollution is the main contributor to elevated indoor PM_1_ levels, while building design, furnishings, and occupant behavior influence how effectively outdoor particles enter and remain indoors, pointing to opportunities for both outdoor emission reduction and building-level interventions.

Tree-based ensemble models were also preferred over more complex deep learning architectures because they provide strong predictive performance on small tabular datasets while retaining a higher level of interpretability, which is essential for understanding environmental drivers of indoor PM_1_ variability. The results show that several advanced ensemble methods converge to similar predictive performance, which suggests that the remaining prediction error is largely driven by dataset constraints rather than model limitations.

Reducing the number of estimators in the Gradient Boosting Regressor decreased the training performance a from near-perfect fit (R^2^ = 0.9998) to a more realistic value (R^2^ = 0.9754), indicating that the model no longer memorizes the training data, but the validation performance remained stable (test R^2^ = 0.6511), suggesting a reduction in overfitting. Consequently, additional improvements in predictive accuracy would likely require improvements in data quality, feature engineering, or increased dataset size rather than further increases in model complexity.

**Figure 8:**
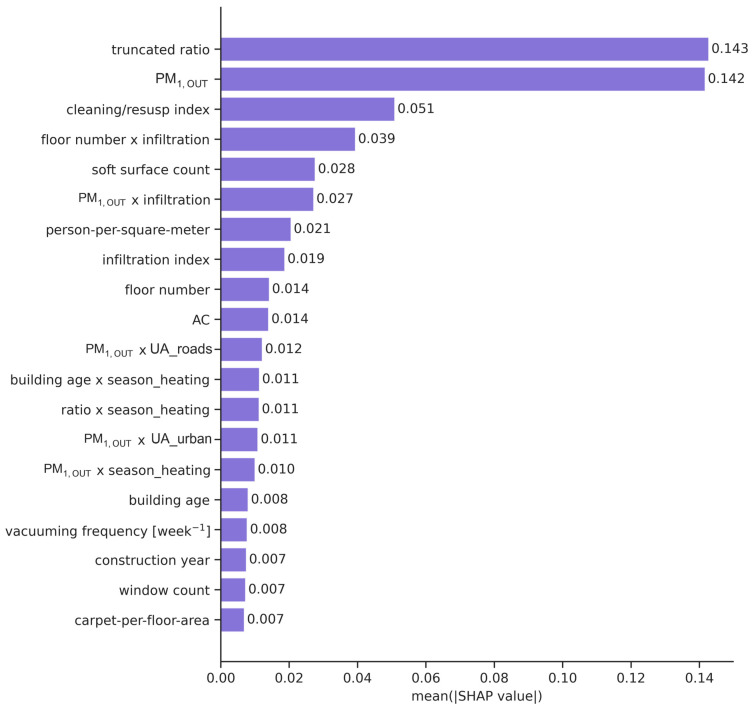
SHAP feature importance for the Gradient Boosting Regressor.

In addition to the intrinsic feature importance metrics inherent to tree-based models, a comprehensive feature examination was conducted, accompanied by the SHAP analysis, to ensure a robust and transparent identification of dominant predictors. The SHAP analysis of the best-performing Gradient Boosting Regressor (Figure 8) showed that outdoor PM_1_ and the truncated ratio were by far the most influential predictors of indoor PM_1_, with mean absolute SHAP values of 0.142 and 0.143, respectively. Secondary contributors included a cleaning/resuspension index, a composite floor number × infiltration variable, a soft surface count, a PM_1,OUT_ × infiltration interaction, and person-per-square-meter density, indicating additional modulation by resuspension, infiltration efficiency, surface reservoirs, and crowding.

**Figure 9:**
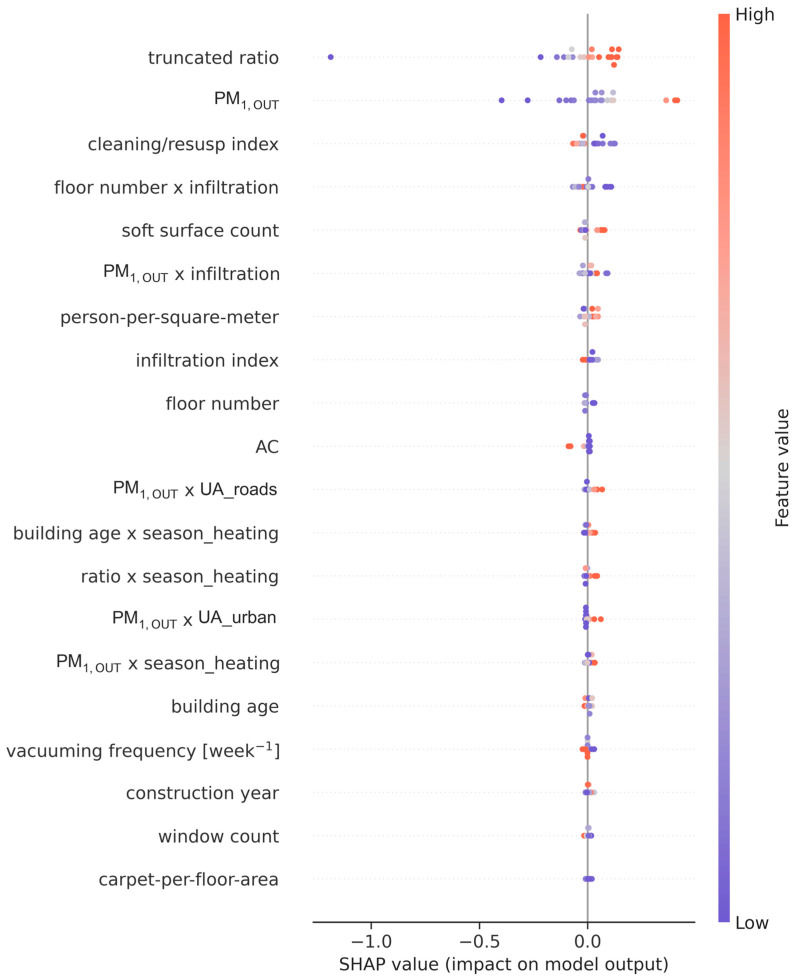
SHAP summary plot for the Gradient Boosting Regressor.

The SHAP summary plot (Figure 9) revealed that higher outdoor PM_1_ and higher truncated ratio values generally increased predicted indoor concentrations, confirming the dominant role of outdoor pollution and infiltration in driving indoor exposure. Elevated cleaning/resuspension scores and greater soft surface counts were also associated with positive SHAP values, supporting the contribution of indoor resuspension processes. In contrast, certain structural and contextual features such as higher floor number, greater building age, and increased carpet-per-floor-area showed more mixed or modest effects, suggesting that their influence operates mainly through interactions with infiltration and outdoor PM_1_ rather than as stand-alone drivers.

Although numerous studies have applied machine learning methods to predict PM_10_ and PM_2.5_, few explicitly include explainable ML approaches such as SHAP analysis [53,82,89,90], and such approaches are even rarer for the PM_1_ fraction. In contrast to time series models that leverage continuous indoor monitoring and rich HVAC or sensor inputs and that can reach R^2^ values above 0.8 for fine particle prediction, most campaign-style studies with sparse indoor sampling and limited contextual information report lower explanatory power. For example, regression models linking indoor PM to outdoor concentrations and a small set of building or behavioral covariates typically explain only a modest fraction of variability, with reported R^2^ often in the 0.3–0.7 range even for PM_2.5_. Within this context, the performance of our PM_1_ models based on 103 paired indoor and outdoor measurements and a limited set of structural and behavioral predictors (test R^2^ ≈ 0.56–0.65) is consistent with and toward the upper end of what is realistically achievable from short residential campaigns without high-frequency indoor sensing or detailed ventilation control data [17,37,47,88,91]. A key strength of this study lies in the use of campaign-based primary measurements conducted in real residential settings rather than relying on secondary datasets or modeled exposure estimates.

While our study provides valuable insights into indoor PM_1_ dynamics using campaign-based primary measurements in real residential settings, a key strength over secondary datasets or modeled estimates several limitations should be noted. The sampling was constrained to weekly measurements to enable subsequent chemical analysis, providing low temporal resolution that likely missed short-term PM_1_ events like cooking or cleaning episodes. Predictor variables derived from participant questionnaires introduce subjectivity and potential recall bias. The measurements were focused solely on living rooms and bedrooms, where children spend most time, excluding other household areas that could influence the overall exposure. Finally, the modest sample size of 103 paired indoor–outdoor observations limits statistical power and model generalizability. Because the holdout test set comprised only ~20 observations (20% of the full dataset), the corresponding test R^2^ values are inherently sensitive to the specific train–test split and should be viewed as an indicative but potentially high-variance estimate of out-of-sample performance. Model selection and performance assessment, therefore, relied primarily on repeated k-fold cross-validation on the training data, which stabilizes performance estimates, although the limited overall sample size still introduces uncertainty in the reported out-of-sample metrics.

## 5. Conclusions

This study shows that in examined households in Zagreb City and Zagreb County (Croatia), indoor PM_1_ concentrations are tightly coupled to ambient levels but exhibit pronounced seasonal and dwelling-specific variability that is toxicologically relevant. During the wintertime, elevated outdoor PM_1_ and stronger indoor-to-outdoor gradients resulted in I/O ratios below unity, indicating that residents were predominantly exposed to particles of outdoor origin even indoors. Multivariate chemometric analysis and clustering demonstrated that this relationship is further modulated by dwelling size and ventilation, the presence of soft furnishings that act as particle reservoirs, occupancy density, cleaning behavior, building age, and heating systems.

Tree-based ensemble machine learning models, specifically the best-performing Gradient Boosting Regressor, reinforced these conclusions by attributing most of the predictive power for indoor PM_1_ to outdoor concentrations, I/O behavior, and variables reflecting infiltration and resuspension processes. Additional improvements in predictive accuracy would likely require improvements in data quality, feature engineering, or increased dataset size rather than further increases in model complexity. From a public health and toxicological perspective, the results highlight that effective reduction in residential PM_1_ exposure cannot rely solely on indoor source control but must combine measures that lower ambient emissions, especially wintertime combustion sources, with building-level interventions such as improved airtightness, filtration, and management of resuspension from carpets and other soft surfaces. Such models could be used population-wide, given the lack of indoor air quality measurements but may rely on available information from questionnaires and ambient air. Hence, it can serve in risk management.

A particular strength of this study lies in the integration of gravimetric indoor and outdoor PM_1_ measurements collected in real residential households with chemometric analysis and explainable machine learning, providing region-specific, exposure-relevant insights grounded in empirical field data, enabling targeted residential IAQ management: optimized ventilation protocols, resuspension mitigation via behavior, and infiltration reduction through retrofits. The use of explainable machine learning strengthens the scientific robustness and trustworthiness of the findings, supporting their relevance for exposure assessment and evidence-based mitigation strategies.

## Figures and Tables

**Table 1:** Descriptive statistics and non-parametric statistics of indoor and outdoor PM_1_ measurements and the indoor-to-outdoor interactions.

	Overall				Non-Heating		Heating		Mann– Whitney U
Metric	*n*	Median [Q1, Q3]	Mean ± SD	Min–Max	*n*	Median [Q1, Q3]	*n*	Median [Q1, Q3]	*p*-Value
PM_1,IN_	103	13.04 [10.27, 17.35]	14.16 ± 6.01	2.48–32.17	53	12.86 [9.96, 15.65]	50	13.47 [10.62, 17.73]	0.41
PM_1,OUT_	103	12.56 [9.87, 17.17]	14.78 ± 7.90	2.11–48.07	53	11.18 [8.63, 13.75]	50	15.94 [11.93, 21.84]	<0.001
PM_1,diff_	103	0.17 [−3.68, 2.45]	−0.62 ± 6.94	−25.23–15.58	53	1.64 [0.16, 3.43]	50	−3.55 [−7.48, 0.27]	<0.001
|PM_1,diff_|	103	3.21 [1.61, 6.42]	4.92 ± 4.92	0.04–25.23	53	1.97 [0.77, 4.79]	50	5.18 [2.32, 10.03]	<0.001
I/O ratio	103	1.02 [0.79, 1.29]	1.12 ± 0.65	0.09–5.75	53	1.15 [1.02, 1.37]	50	0.80 [0.60, 1.02]	<0.001
Truncated I/O	103	1.00 [0.79, 1.00]	0.87 ± 0.20	0.09–1.00	53	1.00 [1.00, 1.00]	50	0.80 [0.60, 1.00]	<0.001

Notes: ${PM}_{1,\;diff}=\;{PM}_{1,\;IN}\;-\;{PM}_{1,\;OUT}$; $I/O\;ratio\;=\;{PM}_{1,\;IN}/{PM}_{1,\;OUT}$; Truncated I/O ratio $I/O=min\left(1,\;{PM}_{1,\;IN}/{PM}_{1,\;OUT}\right).$

**Table 2:** Model evaluation metrics on the training and test set.

	Train				Test			
Model	MAE	MSE	RMSE	R^2^	MAE	MSE	RMSE	R^2^
GBR	0.0504	0.006	0.0775	0.9998	3.3157	19.2284	4.3850	0.6486
CatBoost	0.6652	1.0594	1.0293	0.9610	3.4783	20.0314	4.4756	0.6339
XGB	0.6118	0.9604	0.9800	0.9647	3.6103	23.9732	4.8962	0.5618
AdaBoost	2.1050	7.8870	2.8084	0.7097	4.7352	36.8704	6.0721	0.3261
RF	2.4296	12.1112	3.4801	0.5543	5.0115	41.8474	6.4690	0.2351
DT	2.5349	11.4007	3.3765	0.5804	5.3657	49.7823	7.0557	0.0901

Notes: GBR—Gradient Boosting Regressor; CatBoost—CatBoost Regressor; XGB—Extreme Gradient Boosting; AdaBoost—Adaptive Boosting Regressor; RF—Random Forest Regressor; DT—Decision Tree Regressor.

## Data Availability

The data presented in this study are available upon reasonable request from the corresponding author because the data are not yet being publicly available from the EDIAQI project. This publication has been prepared using the European Union’s Copernicus Land Monitoring Service information; https://doi.org/10.2909/fb4dffa1-6ceb-4cc0-8372-1ed354c285e6 (accessed on 12 January 2026).
